# Editorial: The role of the IGF axis in tumorigenesis and cancer treatment: From genes to metabolites

**DOI:** 10.3389/fendo.2022.1123962

**Published:** 2023-02-14

**Authors:** Guo Mengzhe, Xie Shanshan, Zhao Di, Wu Shihua

**Affiliations:** ^1^Department of Pharmaceutical Analysis, Xuzhou Medical University, Xuzhou, China; ^2^College of Medicine, Zhejiang University, Hangzhou, China; ^3^MD Anderson Cancer Center, University of Texas, Houston, TX, United States; ^4^College of Life Science, Zhejiang University, Hangzhou, China

**Keywords:** IGF, tumorigenesis, genetics, proteomics, metabolomics

The insulin and insulin-like growth factor (IGF) pathway have been documented to play an important role in physiological and pathological biological processes including many types of cancer. Typically, the IGF family has several types of proteins, such as receptors (IGF1R, IGF2R, and IR), ligands (insulin, IGF1, IGF2), ligand-binding proteins (IGFBP1-6), and IGFBP-specific proteases. Increased expression of many components of the IGF family has been invoked in carcinogenesis. The IGF axis has been implicated in many oncogenic processes through activating PI3K/AKT, and MAPK pathways. Moreover, IGF1R has also been associated with cancer metastasis through interacting with the integrin pathway *via* FAK and RACK1. IGF1R appears to be an ideal therapeutic target in many cancers because it is a membrane protein and has receptor tyrosine kinase activity. Two principal types of inhibitors were initially used in trials: monoclonal antibodies IGF1R and small molecule tyrosine kinase inhibitors. However, outcomes in clinical trials were disappointing due to an overall infrequency of objective responses and a lack of predictable biomarkers. Thus, biomarkers should be identified to classify the patient response to anti-IGF treatment.

Omics, including genomics, transcriptomics, proteomics and metabolomics, can provide a prerequisite for a comprehensive understanding of biological systems and mechanisms based on its high-throughput methods leading to a large amount of data. However, the single study for the changes of biomolecules in one certain level has been difficult to meet the rising expectations of systems biology. Therefore, the scientists propose to study the interaction and system mechanism systematically among multi-omics level through biological pathway analysis. In this method, the information of stress disturbance, pathophysiological state or changes after drug treatment can be obtained from genome, transcriptome, proteome, and metabolome experiments and enriched to trace the pathway with the largest and most concentrated changes. Through the analysis of genes, mRNA, proteins, and small molecules *in vivo*, the overall change of substances and molecules were comprehensively analyzed, including the analysis of original pathways and the construction of new pathways, which can reflect the function and metabolic state of tissues and organs, and provide new ideas for exploring the molecular mechanism of biological regulation, key makers and action targets.

In this Research Topic, we aim to encourage the authors finding new mechanisms of the IGF regulating the development of tumors from a clinical and/or molecular biological perspective especially under the integrating analysis of different omics. In the papers accepted after peer review in this Research Topic, the associations between IGF axis and multiple tumorigenesis, prognosis, and drug resistance have been found also with several possible mechanisms. In these papers, van der Velden et al. have identified a set of small molecules that reduces the activities of the growth hormone (GH)/IGF1 axis and its downstream effectors Jak2 and STAT5 by screening a 38480 compounds library. These small molecules also have the potential to inhibit GH-driven cancer growth and anti-cancer drug resistance in breast and colon cancer. Liu et al. used genetics and proteomics method and found that IGF2 receptor mRNA was remarkably upregulated in laryngeal cancer (LC). Moreover, a significant positive correlation between IGF2 receptor expression and tumor microenvironment, immunity, and prognosis. Liu et al. provided a summary analysis and a theoretical basis for the biological role and clinical value of the IGF binding protein (IGFBP) family in low-grade glioma (LGG), and they also found that IGFBP can be considered as the potential drug therapy targets of LGG. Lv et al. have found the association between IGF and lung cancer through large-scale clinical metabolomics. The relevant small molecule metabolites and metabolic pathways were also elucidated.

## Author contributions

All authors listed have made a substantial, direct and intellectual contribution to the work and approved it for publication.

